# Preoperative walking speed and surgical aspects as predictors of early ambulation after abdominal cancer surgery: a prospective cohort study

**DOI:** 10.1007/s00520-026-11072-4

**Published:** 2026-08-19

**Authors:** Gustavo Telles da Silva, Myllena Nassif de Almeida Rohem, Maria Luiza Valério Dalzini, Mônica Maria Pena Quintão, Rodrigo Otavio de Castro Araujo

**Affiliations:** 1 Instituto Nacional de Câncer (INCA), Rio de Janeiro (RJ), Brazil; 2 Universidade Federal Do Rio de Janeiro (UFRJ), Rio de Janeiro (RJ), Brazil

**Keywords:** Cancer, Surgery, Early mobilization, Ambulation

## Abstract

**Purpose:**

To analyze the factors associated with ambulation on postoperative day 1 (POD1) in patients undergoing abdominal cancer surgery. As a secondary objective, the study aimed to investigate the impact of early ambulation on the occurrence of short-term postoperative outcomes.

**Methods:**

This prospective cohort study included patients who underwent elective abdominal cancer surgery between April 2024 and July 2025. Preoperative handgrip strength (HGS) and walking speed, measured using the 10-Meter Walk Test (10MeWT), were assessed preoperatively. Logistic regression was used to identify factors associated with early ambulation.

**Results:**

A total of 122 patients were included in the study, of whom 93 (76.2%) ambulated for the first time on POD1. Multivariate analysis showed that patients with a preoperative walking speed > 0.80 m/s (OR 4.74; 95% CI 1.41–15.96; *p* = 0.01), those who underwent gastrectomy (OR 8.20; 95% CI 1.36–49.32; *p* = 0.02), rectosigmoidectomy (OR 6.99; 95% CI 1.48–32.91; *p* = 0.01), and those with shorter operative times (OR 0.99; 95% CI 0.98–0.99; *p* = 0.02) were independently associated with early ambulation. The median length of hospital stay was significantly shorter among patients who ambulated on POD1 (*p* = 0.01). In-hospital mortality was also lower among those who ambulated on POD1 (*p* = 0.01).

**Conclusion:**

Patients who ambulated on POD1 exhibited a lower frequency of short-term postoperative complications. Preoperative walking speed, operative time, and type of surgical procedure were independent predictors of early ambulation in patients undergoing abdominal cancer surgery.

## Introduction

Due to their high incidence, malignant neoplasms represent a major public health concern worldwide^1^. In 2022, global data estimated approximately 20.0 million new cancer cases and 9.7 million cancer-related deaths [1]. In Brazil, an estimated 704,000 new cancer cases are projected annually for the period 2023–2025 [2].

Cancer treatment requires a multimodal therapeutic strategy that may include chemotherapy, radiotherapy, and tumor resection through surgical procedures [3]. Each year, more than 300 million surgical procedures are performed worldwide [4]. For abdominal cancers, surgical resection often remains the only curative treatment option [5]. Advances in surgical techniques and improvements in perioperative care have made surgery an increasingly feasible and safe treatment option for cancer [5].

In patients undergoing surgery, early mobilization is crucial to counteract the physiological adverse effects of surgical stress and prolonged bed rest [6, 7]. Increased postoperative immobility is associated with prolonged abdominal pain, diarrhea, indigestion, reflux, and delayed passage of the first intestinal gas after surgery [8]. According to the Enhanced Recovery After Surgery (ERAS) concept, early postoperative mobilization is a key component to accelerate recovery after surgery [9–11]. Activities such as sitting at the bedside, standing, in-bed or out-of-bed exercises, and sitting in a chair are encouraged; however, the primary goal is to achieve early ambulation to promote complete recovery [12–14].

Early ambulation is generally defined as independent walking on the first postoperative day and is associated with a reduced risk of postoperative complications, shorter hospital stays, lower healthcare costs, and earlier recovery of independence in activities of daily living [12, 15, 16].

Across different surgical populations, between 48 and 56% of patients with gastrointestinal cancer achieve the scheduled ambulation distance on the first postoperative day; however, a significant proportion experience delayed ambulation, which may adversely affect postoperative outcomes [16, 17]. Currently, there is a knowledge gap regarding predictors that facilitate early ambulation among patients with abdominal cancer undergoing surgery in Latin America. Identifying such predictors may support more individualized perioperative management. Therefore, this study aimed to analyze the factors associated with ambulation on postoperative day 1 (POD1) in patients with abdominal cancer undergoing surgery. As a secondary objective, the study sought to investigate the impact of early ambulation on short-term postoperative outcomes.

## Methodology

### Study design

This prospective cohort study included patients scheduled for abdominal cancer surgery for primary tumor treatment at the surgical wards of a tertiary oncology unit (Hospital do Câncer I, National Cancer Institute, Rio de Janeiro, Brazil) between April 2024 and July 2025. This study was reported in accordance with the Strengthening the Reporting of Observational Studies in Epidemiology (STROBE) guidelines [18]. The study was approved by the Institutional Review Board under report number 6.663.552 (CAAE: 76,711,123.9.0000.5274) on February 22, 2024. This study was performed in line with the principles of the Declaration of Helsinki.. All participants provided written informed consent prior to inclusion.

### Participants

A convenience sample was used for participant recruitment. All consecutive patients aged 18 years or older who underwent elective abdominal cancer surgery lasting longer than three hours were eligible for inclusion. Eligible diagnoses included liver, biliary tract, pancreatic, or gastrointestinal tract cancers (excluding esophageal cancer). Preoperative exclusion criteria were medical contraindication for preoperative assessments, cognitive impairment with inability to understand commands and perform tests, and refusal to participate in the study. After surgery, the exclusion criteria was absence of medical clearance for ambulation on postoperative day POD1.

During hospitalization, all patients were assessed by physiotherapists on POD1. If no clinical contraindications were identified, patients underwent a standard physiotherapy session including posture changes, active mobilization, sitting at the bedside, standing, and walking. Patients who underwent open surgery were instructed to use an abdominal binder during ambulation.

### Outcomes

The primary outcome was the ability to ambulate independently on POD1. Ambulation was considered successful when patients walked more than 20 m [16]. Walking distance was recorded by the physiotherapist who supervised the first ambulation session on POD1.

The secondary outcomes were postoperative complications, postoperative length of hospital stay (LOS), in-hospital mortality, and unplanned 30-day readmission or reoperation. The severity of complications was graded according to the Clavien–Dindo classification (grade II or higher) [19]. All complications were validated by a surgeon (co-author). For patients readmitted or reoperated more than once within 30 days, only the first event was included in the analysis. Readmission and reoperation data were extracted from medical records. LOS was defined as the number of days from surgery to hospital discharge.

### Assessment

#### Clinical and sociodemographic variables

Study data were collected and managed using REDCap electronic data capture tools hosted at National Cancer Institute [20]. Clinical and sociodemographic variables were age, sex, Charlson Comorbidity Index, body mass index (kg/m^2^), neoadjuvant chemotherapy, American Society of Anesthesiologists (ASA) physical status classification (I–II/III–IV), type of surgical procedure, surgical approach, use of epidural anesthesia, operative time (minutes), estimated blood loss (mL), and walking distance on POD 1 (meters).

#### Physical capacity assessment

Preoperative physical performance was evaluated using the following assessments: handgrip strength (HGS), measured with a handgrip dynamometer [21], and walking speed, assessed by the 10-Meter Walk Test (10MeWT) [22]. Tests were performed in the surgical ward the day before surgery. All evaluations were performed in the same order by trained physiotherapists. During the assessments, patients were monitored, and rest periods between tests were strictly observed, ensuring at least 3 min of rest between each evaluation.

HGS was assessed using a hydraulic hand dynamometer (Model 60,440, Jamar®, Bolingbrook, IL, USA). All measurements were performed with the participant seated, shoulder adducted and in a neutral rotation, elbow flexed at 90°, forearm in a neutral position, and wrist in a neutral position [21]. The highest value obtained from three attempts of maximal voluntary contraction, performed at 1-min intervals, was used for analysis [21].

For the 10MeWT, participants were instructed to walk along a hospital corridor at their usual pace, with timing performed using a stopwatch. The first 1.2 m of the course allowed for gait acceleration and were not included in the recorded speed. The subsequent 10 m constituted the measured distance, and the final 1.2 m allowed for deceleration, with no timing recorded. Three trials were conducted to minimize learning effects, and the best performance was used for data analysis [22]. Walking speed was calculated using the formula *v* = *Δs/Δt*, where *Δs* represents distance (meters) and *Δt* represents time (seconds). Walking slowness was defined as a gait speed ≤ 0.80 m/s [23, 24].

### Statistical analysis

Data normality was verified using the Kolmogorov–Smirnov test. Continuous variables were presented as mean ± standard deviation (SD) or median and interquartile range (IQR, 25–75th percentile), according to data distribution. Categorical variables were expressed as absolute frequencies and percentages. Group comparisons (ambulated vs. non-ambulated on POD1) were performed using the chi-square test or Fisher’s exact test for categorical variables, and t-test or Mann–Whitney U test for continuous variables, as appropriate. The Kaplan–Meier method was used to analyze the association between ambulation status and time to hospital discharge, with differences between curves assessed using the log-rank test. To identify factors independently associated with early ambulation, binary logistic regression analysis was performed. Variables with *p* < 0.15 in univariate analyses were entered into the multivariate model [25]. Collinearity among predictors was assessed before model inclusion. Statistical significance was set at *p* < 0.05. All analyses were conducted using SPSS Statistics for Windows, version 21.0 (IBM Corp., São Paulo, Brazil).

## Results

### Patient population

**Fig. 1:**
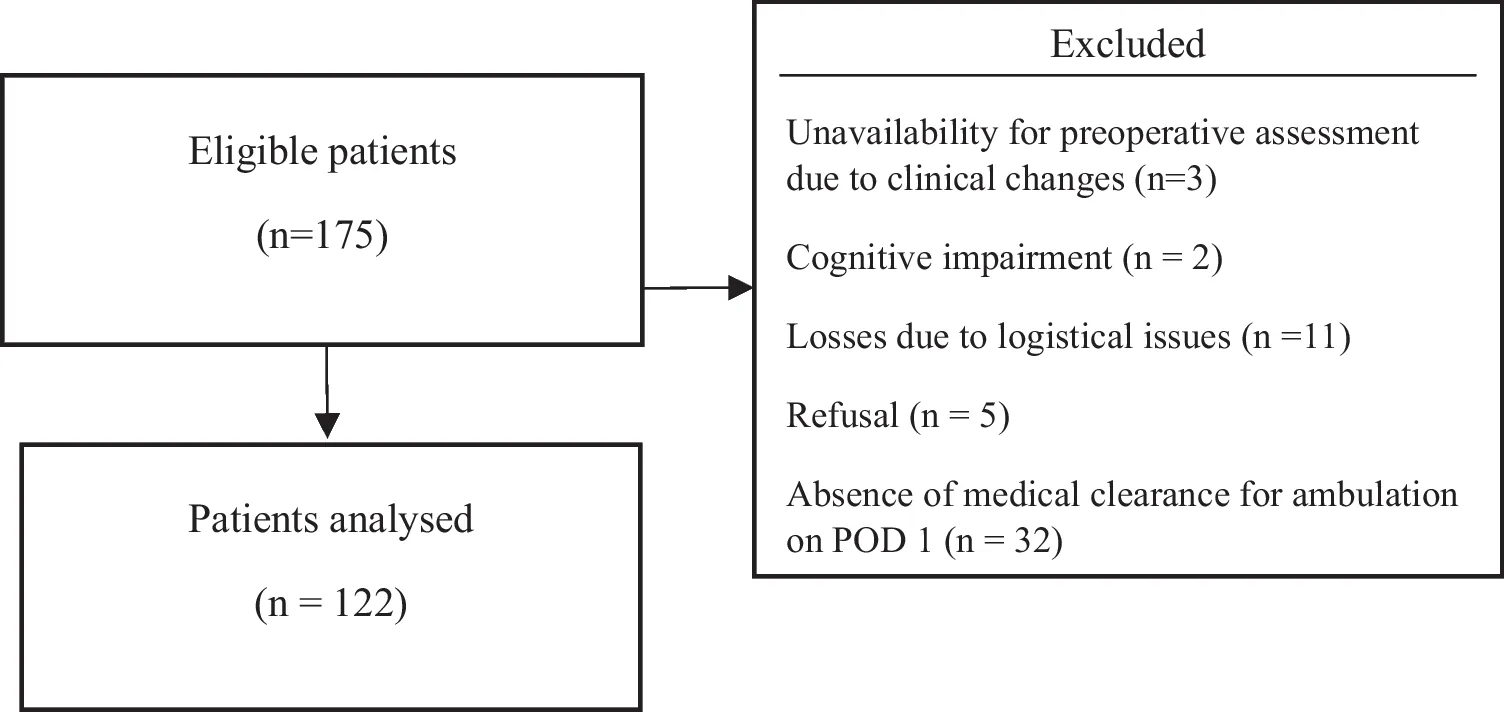
Flowchart of the study

A total of 175 patients were admitted to the surgical ward during the preoperative period for surgery to treat a primary gastrointestinal tumor. After applying the eligibility criteria, 122 patients were included in the study Fig. 1. The median age was 64 years (58–71). Most patients were female (64.8%), classified as ASA I–II (88.5%), and had a median BMI of 26 kg/m^2^ (23–30). The median preoperative handgrip strength (HGS) was 17.5 kgf (10–23), and 83.6% of the patients presented a preoperative walking speed greater than 0.8 m/s. Regarding the surgical procedure, rectosigmoidectomy was the most frequent (30.3%), followed by colectomy (24.6%) and gastrectomy (17.2%). The most common surgical approach was open surgery (53.3%), followed by laparoscopic (38.5%), with a median operative time of 240 min (180–300) Table 1.

**Table 1:** Patient characteristics (*n* = 122)

Characteristics	Full cohort
Age (Years)	64 (58–71)
Gender, n (%)	
Male	43 (35.2)
Female	79 (64.8)
BMI (kg/m^2^)	26 (23–30)
CCI, n (%)	
≤ 3	104 (85.2)
> 3	18 (14.8)
Neoadjuvant Chemotherapy, n (%)	
Yes	23 (18.9)
No	99 (81.1)
ASA class	
1 −2	108 (88.5)
3–4	14 (11.5)
Preop HGS (kg)	17.5 (10–23)
Preop 10MeWT (m/s)	
≤ 0.80	20 (16.4)
> 0.80	102 (83.6)
Surgical Procedures, n (%)	
Gastrectomy	21 (17.2)
Rectosigmoidectomy	37 (30.3)
Colectomy	30 (24.6)
Anterior Rectal Resection	20 (16.4)
Others	14 (11.5)
Operative approach, n (%)	
Open	65 (53.3)
Laparoscopic	47 (38.5)
Robotic	10 (8.2)
Epidural anaesthesia	47 (38.5)
Operative time, (min)	240 (180–300)
Estimated blood loss (mL)	62.4 (105.8)

Values are mean ± SD, median (IQR), except where indicated

*IQR* Interquartile range, *BMI* Body Mass Index, *CCI* Charlson Comorbidity Index, ASA American Society of Anesthesiology, *WHO* World Health Organisation, *Preop 10MeWT* 10-Meter Walk Test, *Preop HGS* Preoperative Handgrip Strength

### Postoperative first deambulation

A total of 93 patients (76.2%) performed their first ambulation on postoperative day 1 (POD1). The median walking distance during the first ambulation was 90 m (50–98). In contrast, 29 patients (23.8%) were unable to ambulate on POD1. The reasons for delayed ambulation included hypotension (*n* = 14), inadequate postoperative pain management (*n* = 9), inadequate control of postoperative nausea and vomiting (*n* = 3), lower limb weakness (*n* = 1), and patient refusal (*n* = 2).

### Factors associated with early ambulation

In the univariate analysis, sex, preoperative HGS, preoperative 10-m walking test (10MWT), and surgical procedure were associated with early ambulation on POD1 Table 2. Variables with *p* < 0.15 in the univariate analysis were included in the multivariate logistic regression. The logistic regression model was statistically significant (x^2^(16) = 28.42; *p* < 0.001), demonstrated good fit according to the Hosmer–Lemeshow test (*p* = 0.92), explained 31% of the outcome variance (Nagelkerke R^2^), and correctly classified 82% of the cases. Patients with a preoperative walking speed > 0.8 m/s were 4.7 times more likely to achieve early ambulation on POD1 compared with those with a walking speed ≤ 0.8 m/s (OR 4.70; 95% CI 1.36–16.32; *p* = 0.02). Patients who underwent gastrectomy were 8.2 times more likely to achieve early ambulation (OR 8.20; 95% CI 1.36–49.32; *p* = 0.02), and those who underwent rectosigmoidectomy were 6.9 times more likely (OR 6.99; 95% CI 1.48–32.91; *p* = 0.01). Additionally, a shorter operative time was independently associated with early ambulation (OR 0.99; 95% CI 0.98–0.99;* p* = 0.02) Table 3.

**Table 2:** Factors associated with postoperative early ambulation (univariate analysis)

	Early Ambulation			
Characteristics	Yes (*n* = 93)	No (*n* = 29)	OR (95% CI)	*p* value
Age (Years)	64 (58–71)	64 (56–70)	1.01 (0.97–1.05)	0.40
Sex, *n* (%)				
Male	38 (40.9)	5 (17.2)	3.31 (1.16–9.46)	**0.02**
Female	55 (59.1)	24 (82.8)		
BMI (kg/m^2^)	25 (22–30)	27 (24–31)	0.97 (0.90–1.04)	0.45
CCI, n (%)				
> 3	16 (17.2)	2 (6.9)	2.80 (0.60–13.00)	0.18
≤ 3	77 (82.8)	27 (93.1)		
NC, *n* (%)				
No	77 (82.8)	22 (75.9)	1.53 (0.56–4.19)	0.40
Yes	16 (17.2)	7 (24.1)		
ASA class, *n* (%)				
1–2	84 (90.3)	24 (82.8)	1.94 (0.59–6.35)	0.27
3–4	9 (9.7)	5 (17.2)		
Preop HGS (kg)	18 (12–24)	12 (8–20)	1.05 (1.00–1.11)	**0.03**
Preop 10MeWT (m/s)				
> 0.80	84 (90.3)	18 (62.1)	5.70 (2.06–15.7)	**0.001**
≤ 0.80	9 (9.7)	11 (37.9)		
Surgical Procedures, *n* (%)				
Gastrectomy	18 (19.4)	3 (10.3)	8.00 (1.58–40.29)	**0.01**
Rectosigmoidectomy	31 (33.3)	6 (20.7)	6.88 (1.74–27.18)	**0.006**
Colectomy	24 (25.8)	6 (20.7)	5.33 (1.33–21.32)	**0.01**
Anterior Rectal Resection	14 (15.1)	6 (20.7)	3.11 (0.74–12.95)	0.11
Others	6 (6.5)	8 (27.6)		
Operative approach, *n* (%)				
Laparoscopic	37 (39.8)	10 (34.5)	1.41 (0.58–3.43)	0.44
Robotic	9 (9.7)	1 (3.4)	3.44 (0.40–29.18)	0.25
Open	47 (50.5)	18 (62.1)		
Operative time, (min)	240 (180–300)	270 (227–360)	0.99 (0.99–1.00)	0.05
Estimated blood loss, (mL)	55.7 (93.7)	84.1 (137.5)	0.99 (0.99–1.00)	0.21
Epidural anaesthesia				
Yes	34 (68.0)	13 (65.0)	1.14 (0.38–3.41)	0.80
No	16 (32.0)	7 (35.0)		

Values are mean ± SD, median (IQR), except where indicated

*IQR* Interquartile Range, *BMI* Body Mass Index, *CCI* Charlson Comorbidity Index, *NC* Neoadjuvant chemotherapy, *AS*A American Society of Anesthesiology, *WHO* World Health Organisation, *Preop 10MeWT* 10-Meter Walk Test, *Preop HGS* Preoperative Handgrip strength. Bold figures represent statistically significant values

**Table 3:** Factors associated with postoperative early ambulation (multivariate analysis)

Characteristics	OR (95% CI)	*p* value
Male	2.37 (0.55–10.28)	0.24
Preop HGS (kg)	1.02 (0.95–1.10)	0.42
Preop 10MeWT (> 0.80 m/s)	4.74 (1.41–15.96)	**0.01**
Operative time, (min)	0.99 (0.98–0.99)	**0.02**
*Surgical Procedures*		
Gastrectomy	8.20 (1.36–49.32)	**0.02**
Rectosigmoidectomy	6.99 (1.48–32.91)	**0.01**
Colectomy	4.62 (0.95–22.35)	0.05
Anterior Rectal Resection	3.98 (0.76–20.61)	0.10
Others		

Values are mean ± SD, median (IQR), except where indicated

*Preop 10MeWT* 10-Meter Walk Test, *Preop HGS* Preoperative Handgrip strength. Bold figures represent statistically significant values

### Postoperative outcomes

**Fig. 2:**
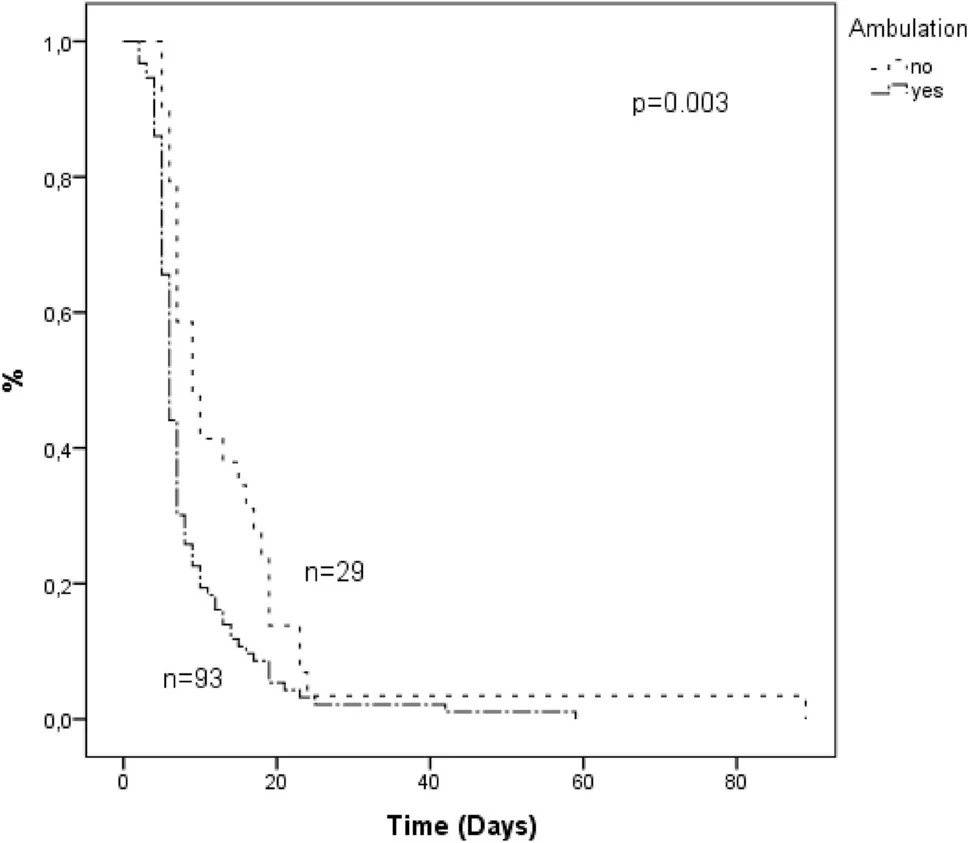
Postoperative length of stay

The median postoperative length of stay (LOS) was 6 days (95% CI 5.53–6.46) for patients who ambulated on POD1 and 9 days (95% CI 5.83–12.16) for those who did not, with a statistically significant difference (*p* = 0.003) Fig. 2. Postoperative complications classified as Clavien–Dindo grade ≥ II were more frequent among patients who did not ambulate on POD1 (41.4% vs. 19.4%; *p* = 0.01), and in-hospital mortality was also higher in this group (10.3% vs. 0%; *p* = 0.01) Table 4.

**Table 4:** Postoperative outcomes (*n* = 122)

	Early Ambulation Yes (*n* = 93)	Early Ambulation No (*n* = 29)	*p* value
Clavien ≥ 2, N (%)	18 (19.4)	12 (41.4)	0.01
Median LOS (days), (IQR)	6 (5.53–6.46)	9 (5.83–12.16)	0.003
In-hospital mortality, N (%)	0 (0.0)	3 (10.3)	0.01
30-day Readmission, N (%)	14 (15.1)	2 (6.9)	0.21
30-day Reoperation, N (%)	6 (6.5)	0 (0.0)	0.18

Values are median (IQR), except where indicated

*LOS* Length of stay

## Discussion

Abdominal cancer patients who underwent surgery for treatment of the primary tumor and achieved early ambulation presented a lower frequency of postoperative complications and shorter hospital stay. Walking speed, operative time, and type of surgery were associated with early postoperative ambulation.

The transfer of patients undergoing surgery for cancer treatment to surgical wards is a complex process that requires continuous monitoring and trained professionals. The *Enhanced Recovery After Surgery* (ERAS) guidelines advocate early postoperative mobilization to counteract catabolic changes caused by immobilization and to preserve muscle strength. Early ambulation is strongly recommended within the ERAS framework [26]. Although some institutions have not fully implemented ERAS protocols, early mobilization has become a universal practice in surgical care worldwide [13].

Identifying modifiable factors associated with delayed ambulation may facilitate the early recognition of vulnerable patients and enable personalized perioperative care. In this study, 76.2% of patients achieved their first ambulation on postoperative day 1 (POD1), and preoperative walking speed, type of surgical procedure, and operative time were independent predictors of early ambulation. Longer operative duration and procedures involving multiple organs were associated with delayed ambulation, likely reflecting greater surgical stress and slower postoperative recovery. Similar findings have been reported in other observational studies examining intraoperative factors [27, 28]. A retrospective study of 217 patients who underwent abdominal surgery demonstrated that a higher neutrophil-to-lymphocyte ratio (OR 1.11; 95% CI 1.01–1.23), ASA class III (OR 4.99; 95% CI 1.15–21.5), and longer operative duration (OR per 1 h = 1.27; 95% CI 1.06–1.43) were independently associated with the inability to ambulate without assistance on POD1 [27]. A prospective study conducted in Ethiopia evaluated 422 patients who underwent major abdominal surgery and found that operative time (OR 3.96; 95% CI 1.87–8.38), intraoperative blood loss (OR 2.68; 95% CI 1.24–5.79), and the volume of intravenous fluids administered (OR 2.54; 95% CI 1.03–6.24) were independently associated with delayed postoperative ambulation [28]. Consistent with the present study, these studies did not demonstrate an independent association between delayed postoperative ambulation following abdominal surgery and the operative approach. These findings support the hypothesis that longer surgical duration, which is associated with greater anesthetic exposure, increased cumulative tissue trauma, and a higher likelihood of intraoperative hemodynamic instability, may represent a more robust predictor of delayed ambulation than the surgical technique itself.

Other studies have highlighted the importance of functional status in predicting early ambulation after abdominal surgery [16, 29, 30]. Nishijima et al. (2020) [16] retrospectively analyzed 718 patients who underwent general and digestive surgery and found that an ECOG-PS of 2–4, higher CONUT score, non-laparoscopic procedures, and transvenous opioid use were independent predictors of delayed ambulation. A multicenter prospective study that investigated early ambulation after laparoscopic gastrointestinal tumor surgery in 427 patients reported sex-specific results: among men, lower preoperative handgrip strength (OR = 1.109, *p* < 0.001) and a higher number of indwelling tubes (OR = 0.666, *p* = 0.006) were risk factors; among women, lower handgrip strength (OR = 1.148, *p* = 0.001) and prolonged operativetime (OR = 0.587, *p* = 0.001) predicted delayed ambulation [30]. In the present study, preoperative handgrip strength was an independent predictor for early ambulation only in the univariate analysis.

In the current study, preoperative walking speed was an independent predictor of early ambulation in patients with abdominal cancer. Recently, there has been growing interest in the objective assessment of walking speed at a usual pace [31, 32]. Walking speed is widely recognized as an indicator of functional status, overall physical performance, cardiovascular health, and the ability to perform daily activities [23, 33]. According to the International Academy on Nutrition and Aging, slow walking speed may be indicative of sarcopenia and frailty, and it can predict mortality as well as the occurrence of medical complications such as hospitalization, disability, falls, and cognitive decline [23, 33].

The walking test is simple, inexpensive, safe, and demonstrates excellent test–retest reliability among patients with cancer. It is widely used in clinical practice to assess usual gait performance [31, 32, 34]. The preoperative identification of patients with slow walking speed may be valuable in two ways. First, in low- and middle-income countries or institutions without formal prehabilitation programs, the 10MWT could be implemented during hospital admission to identify patients with reduced functional capacity and ensure systematic peri- and postoperative multidisciplinary care. Second, it enables early recognition of high-risk individuals who could benefit from a prehabilitation program focused on perioperative nutritional support and physical exercise to improve mobility and reduce postoperative complications. Traditionally, oncology patients seeking to regain functional independence in the hospital setting have benefited from rehabilitation programs [35]. In recent years, interest in prehabilitation among patients with digestive system cancers has grown [36, 37]. Multimodal prehabilitation programs—combining resistance exercise, aerobic training, and nutritional support—have demonstrated efficacy in improving the physical and functional capacity of patients scheduled for abdominal surgery [36, 37]. Notably, these interventions may impact modifiable risk factors such as reduced handgrip strength and slow gait speed.

The benefits of early ambulation after abdominal surgery are well documented [26, 38, 39]. However, studies focusing exclusively on cancer patients remain scarce in Latin America. In this Brazilian study, patients who ambulated on POD1 had a shorter postoperative length of stay, lower frequency of Clavien–Dindo grade ≥ II complications, and lower in-hospital mortality. These findings are consistent with previous reports from Asia, Europe, and North America [26, 27, 39]. For instance, a Japanese study of 217 patients after abdominal surgery demonstrated that inability to ambulate without assistance on POD1 was associated with prolonged hospitalization (median 21 [15–39] vs. 13 [11–22] days, *p* < 0.001) [27]. An American study of 108 elective abdominal surgery patients showed that delayed ambulation was associated with a higher rate of postoperative complications (16% vs. 1%, *p* < 0.01) [39]. A Swiss cohort study of 676 patients reported that 58% did not meet the ERAS target of mobilization for at least 6 h on POD1, and delayed mobilization was associated with higher overall (Clavien grades I–V, 55% vs. 29%, *p* < 0.001) and major complication rates (Clavien IIIb–V, 16% vs. 7%, *p* < 0.001), as well as longer hospital stay (12 ± 14 vs. 6 ± 7 days, *p* < 0.001) [26]. Conversely, Nishijima et al. (2020) [16] found no difference in morbidity between patients who ambulated on POD1 and POD2, though those who began ambulation after POD3 had significantly higher rates of complications. Calotta et al. (2018) [40], in a study of patients undergoing colorectal oncologic resection with perineal reconstruction, showed that early ambulation was a significant predictor of reduced minor complications (OR 0.16; 95% CI 0.03–0.69), although no protective effect was found for major complications. A systematic review and meta-analysis assessing the effects of early postoperative mobilization after gastrointestinal surgery demonstrated that early mobilization accelerated gastrointestinal recovery but did not significantly reduce morbidity, improve postoperative mobility, or shorten hospital stay [41]. Notably, this meta-analysis did not include cancer-only populations. Finally, a Swedish study found no association between early mobilization and 30-day readmission following abdominal cancer surgery [42].

This study has some limitations. The relatively small sample size may have introduced type II error. Furthermore, the heterogeneity of the surgical procedures performed may have introduced variability in the outcomes, limiting direct comparisons across diferente surgical subgroups. Additionally, the generalizability of the findings is limited as this was a single-center study conducted in a hospital with established protocols, including scheduled physiotherapy sessions starting on POD1. These structured interventions may have positively influenced early ambulation rates. Nevertheless, to the best of our knowledge, this is the first study in Latin America to provide data on preoperative physical performance and muscle strength in a selected group of abdominal cancer patients.

## Conclusions

In conclusion, early ambulation on POD1 was associated with a lower frequency of short-term postoperative complications and shorter hospital stay. Preoperative walking speed, operative time, and type of surgery (gastrectomy and rectosigmoidectomy) were independent predictors of early ambulation among abdominal cancer patients. Larger, multicenter studies are warranted to strengthen the level of evidence and further validate these findings.

## Data Availability

No datasets were generated or analysed during the current study.

## Declarations

The study was approved by the Research Ethics Committee of the Brazilian National Cancer Institute (protocol CAAE: 76711123.9.0000.5274, approval number 6.663.552/2024). This study was performed in line with the principles of the Declaration of Helsinki. All participants signed free and informed consent forms.
